# Supplementary material for: Swedish private-sector employees’ experiences of promoting and hindering factors for working while having mental health problems: A qualitative study
Source: PLoS One. 2026 Feb 18;21(2):e0342773. doi: 10.1371/journal.pone.0342773 (PMC12915946; doi:10.1371/journal.pone.0342773)
Supplement: S1 File — (DOCX) [file pone.0342773.s001.docx]

About the Interview guide: The guide is structured in query areas with facilitating and hindering factors at the workplace and on an individual level. The introductory question is open and broad, after which there are suggestions for questions to ask if the informant has difficulty understanding or relating to the question area.

**Introduction**

- Would you like to start by telling me a little bit about yourself?
- Tell me about your work! What does a normal working day look like?
- Is it different from 1 year ago?

**Facilitating and hindering factors for staying at work.**

In this interview, I am interested in how you experienced working from the time you enrolled in the study, about 1 year ago, until today. When answering the questions, base your responses on your own experiences.

So, if we delve deeper into what promoted or hindered staying at work.

At the workplace

- If you think about the workplace, was there anything that made it easier to stay at work?
- Did you feel that there was something that made it difficult to stay at work?
- If you think about your colleagues?
  - Expectations from colleagues?
  - Support from colleagues?
- If you think about your manager?
  - Expectations from manager?
  - Support from manager?
- Was there anything in the atmosphere at the workplace that promoted or hindered staying at work?
- If you think about the workplace in general, were there possibilities for job adaptations that made it easier to continue working (time, tasks, other)?
- Being able to work undisturbed can be a prerequisite for working – is that right for you?
- Sometimes it can be difficult to start or finish tasks, concentrate, manage social contacts etc. Is this true for you? Was there anything in the workplace that made it easier to handle?

Individual

- If you think about yourself and your own resources, is there anything that you feel has affected your ability to stay at work?
  - Hindered?
  - Facilitated?
- Did you use any specific strategies to be able to stay at work?
- How did you experience the balance between work and private life?
  - Was there anything in your private life that you felt made it difficult for you to stay at work?
  - Was there anything in your private life that you found facilitated staying at work?
- Were there any habits or routines that have been important to you to be able to stay at work?
- Did you experience any symptoms that hindered staying at work (Difficulty to concentrate, start or finish tasks, manage social contacts, headaches)?
- What about recovery…
  - In everyday life?
  - At work?
  - Do you think this affected your ability to work during this period?
- Is there anything else that I haven't asked about that you would like to add about what facilitated or hindered staying at work?

**Example of prompts to use during the interview:**

- When you say ‘…’, what do you mean?
- You told me earlier about... Can you elaborate on that?
- Can you give an example of...?
- Summarize and reflect what the informant says.
