# Supplementary material for: Swedish private-sector employees’ experiences of promoting and hindering factors for working while having mental health problems: A qualitative study
Source: PLoS One. 2026 Feb 18;21(2):e0342773. doi: 10.1371/journal.pone.0342773 (PMC12915946; doi:10.1371/journal.pone.0342773)
Supplement: Attachment — Submitted filename: Response to Reviewers.docx [file pone.0342773.s003.docx]

Dear Editor,

Thank you for the possibility to revise and resubmit our manuscript with the title “Swedish private-sector employees’ experiences of facilitators of, and barriers to, staying at work with mental health problems: a qualitative study.”, submission ID PONE-D-25-25048. We would also like to thank you and the reviewers for your valuable comments. We believe these have contributed to improving our manuscript greatly. You will find our point-by-point response below. Changes in the manuscript are indicated with row number in the point-by-point response.

On behalf of the authors,

Yours sincerely,

Anna Frantz

Additional Editor Comments:

The following points should be addressed:

1. Introduction

The phrase "facilitators of, and barriers to" in the title essentially refers to identifying promoting and hindering factors. Therefore, I suggest revising the wording for clarity and conciseness.

Response: We agree with your suggestion of replacing the wording “facilitators of, and barriers to” and have replaced this with promoting and hindering factors throughout the document.

The introduction requires clearer organization. The explanation regarding the PRIME study would be more appropriately placed in the Methods section, particularly under participant recruitment.

Response: Regarding the description of the PRIME-study, we have moved this to the methods section, line 142-146.

Although the authors provide a theoretical framework for defining "staying at work" (SAW), this section currently reads more like a conclusion rather than a theoretical positioning. The subheading used for this section should be removed, and the discussion should instead be integrated into the main flow of the introduction. In addition, the conceptual distinction between SAW and presenteeism remains unclear and should be explicitly addressed.

Response: We have removed the sub-heading for the section ‘Theoretical underpinnings’ and integrated the description of theoretical frameworks into the introduction (line 67-68). With regards to the conceptual distinction between SAW and presenteeism, we find that it would fit better in the discussion, line 504-515.

It is also important to more clearly articulate the rationale and unique contribution of this study within the existing body of research.

Response: We have added a clarification of the study’s unique contribution in relation to previous studies, line 88-89 in the introduction.

2. Methods

As a qualitative study, greater methodological rigor is needed. Qualitative research demands that the researcher maintain objectivity, demonstrate contextual understanding of participants, and ensure the trustworthiness of the findings.

First, the limited number of participants raises questions about whether the study achieved sufficient data saturation. A more cautious interpretation of the results is warranted, and the limitations should be clearly acknowledged.

Response: Regarding the number of participants – we agree that it raises questions about whether enough data was collected. We have extended the discussion of limitations related to the number of participants on line 477-484. It is possible that a certain group was self-selected into this study – the additional information about the study participants included in the results section allows for a more nuanced interpretation of the transferability of the findings and the possible limitations of the study group.

We have reformulated the concluding remarks to make a more cautious interpretation of the results, line 523-532.

Second, additional information is needed regarding the data analysis process. Specifically, how many researchers were involved in analyzing the interview data, and what procedures were followed to ensure analytical reliability ?

Response: A clarification of how the authors contributed to the data analysis process, as well as a clarification of the procedure to ensure analytical reliability, is provided on lines 201, 203-207.

(The final sample of 18 participants was drawn from a larger pool within the PRIME study. Rather than detailing the selection process, this could be briefly summarized, with the emphasis placed on describing the final sample and its characteristics . Demographic and occupational details of participants should be included in the Results section.

Response: We have summarized the selection process more briefly in the methods section and removed redundant information (line 168-170). A table with participant demographics has been added to the results section (line 226).

3. Other Points

Abbreviations such as AFr, EBB, IA, and AFi should be written out in full upon first use.

Response: We have written out these abbreviations upon first use (lines 177-179) and reviewed all abbreviations throughout the manuscript.

The manuscript currently lacks information about participant characteristics, which should be clearly presented.

Response: A table with participant characteristics has been added (line 226)

Reviewer 1

Reviewer #1: Thank you for the opportunity to review your manuscript, which addresses a timely and important topic: the experiences of private-sector employees with mental health problems in relation to staying at work (SAW). Your study contributes to the understanding of workplace mental health by focusing on facilitators and barriers from the employee perspective, particularly in blue- and pink-collar occupations, a population often underrepresented in such research.　　The manuscript is well organized and clearly written. The use of reflexive thematic analysis is appropriate, and the application of the JD-R and IGLOO frameworks offers valuable potential for interpretation. However, to enhance the methodological rigor and interpretive strength of your study, the following major revisions are recommended:

Major Points

1. Sample Representativeness and Self-selection Bias

The study included 18 interview participants from a pool of 129 eligible individuals (14% response rate). While small samples are acceptable in qualitative research, this low participation rate introduces concerns about potential self-selection bias and limits the transferability of the findings . Please discuss this limitation more explicitly in the Methods and Discussion sections.

Response: Thank you for commenting on this. We have considered your suggestion and extended the discussion regarding our sample and transferability in the methodological discussion (lines 477-484).

2. Participant Mental Health Status and Heterogeneity

While GHQ-12 and a predictive item on sickness absence were used for inclusion, there is limited discussion of the severity or heterogeneity of participants' mental health problems . More information or reflection on this point would provide helpful context for interpreting the findings.

Response: Thank you for this suggestion. We have added information about symptom levels of anxiety and depression as measured with the Hospital anxiety and depression scale at 12 month follow-up corresponding to the time of the interview (Table 1, line 226). We have also extended the methodological discussion with regard to symptom severity (line 477-484), see text extract below.

“The interview was conducted one year after the baseline measurement, and symptoms have likely changed during the year between baseline and the interview. The interview questions were posed in a retrospective manner to elicit participant’s experiences from the year preceding the interview. Participants with more symptoms may find it too burdensome to participate in interviews which may limit transferability of findings to these individuals. Additionally,most participants in the study were born in Sweden, which may limit the transferability of findings to employees born outside Sweden or Europe”

3. Theoretical Integration

Although the manuscript references both the JD-R and IGLOO frameworks , their integration into the analysis appears superficial. The study would benefit greatly from a clearer mapping between themes and theoretical constructions. Consider adding a summary table or diagram to illustrate how each identified theme relates to specific elements of the frameworks.

Response: Thank you for this comment. Our intention was to use the IGLOO framework to identify on what level each promoting/hindering factor was situated on – individual level, group level, leader level, organizational level or overarching level. The JD-R is not used specifically in the data analysis, as the data analysis was inductive and not guided by a theory. The JD-R was used as a framework to discuss our results. We have extended the discussion of our results in relation to the JD-R (lines 433-443) and clarified our use of the IGLOO framework in Table 2 (line 348)

4. Conceptual Clarity: SAW vs. Presenteeism

The terms “staying at work” and “presenteeism” are used somewhat interchangeably, yet they are conceptually distinct. Please clarify how you define and distinguish these concepts in the context of your study or justify their use as overlapping constructs .

Response: We agree that there is room for improvement in the description of the concepts. We have added a clearer definition for staying at work in the introduction (line 67-68 ) After careful consideration, we have decided to move the section concerning presenteeism to the discussion to improve the clarity of the introduction. (line 505-516)

Minor Suggestions

5. Language and Tone:

The manuscript is generally written in clear and standard English. However, a few expressions may benefit from refinement for tone and clarity. For instance, consider replacing "battered" (p. XX) with a more neutral term like "exhausted" or "fatigued."

Response: We have reviewed the manuscript with consideration of tone and clarity. We have replaced the wording “battered” with exhausted (line 345).

6. Use of Visual Aids:

Figure 1 and Figure 2 are helpful but could benefit from additional explanation in the main text to maximize their contribution to the reader’s understanding.

Response: We have added additional explanation in the text for Figure 1, lines 211-216, and Figure 2, line 229.

7. Abbreviations:

Consider providing a list of abbreviations (e.g., SAW, MHPs, JD-R ) for reader reference and ensure that all terms are defined upon first use.

Response: Thank you for this suggestion! We have added a list of abbreviations at the end of the manuscript and defined terms upon first use.

Conclusion

In sum, this is a promising and well-conceived manuscript that addresses a significant issue in occupational health. With revisions to strengthen the theoretical grounding, clarify key concepts, and address methodological limitations, the manuscript will be considerably improved and may be suitable for publication in PLOS ONE.

Response: Thank you for your encouraging words.

Reviewer #2: Thank you for the opportunity to review the manuscript titled “Swedish private-sector employees’ experiences of facilitators of, and barriers to, staying at work with mental health problems: a qualitative study.” The conclusions you draw are highly important for future academic debate in occupational health and represent a valuable contribution. However, there are still several points that need clarification:

Major comments

1. Adverse job stressors that negatively affect mental health have already been well established in prior research. Your study is predicated on the idea that employees with mental health problems face different factors, yet the theoretical basis for why these factors should differ needs to be reinforced. Please strengthen the Introduction with a clear rationale for why these alternative factors are expected in this population.

Response : Thank you for your valuable comment. The rationale behind our study is the lack of knowledge on how employees with mental health problems experience what hinders or promotes stay at work, especially in blue collar sectors. We have added a clarification of the study’s unique contribution in relation to previous studies, line 88-89 in the introduction.

2. Lines 84–92 do not belong in the Introduction and should be relocated to the Methods section

Response: We have moved this section from the introduction to methods (line 142-146)

3. Isn’t “staying at work ” essentially the inverse of job leave (turnover)? Please clarify how your exploration of “stay at work” differs from existing studies that examine predictors of job leave.

Response: Thank you for raising this question. We have added a clarification to the definition of staying at work (line 67-68) and extended the discussion (lines 505-516). To the best of our knowledge, staying at work is not necessarily the inverse of job leave or turnover, as it potentially could be reported regardless of changing workplace or not.
