# Supplementary material for: Swedish private-sector employees’ experiences of promoting and hindering factors for working while having mental health problems: A qualitative study
Source: PLoS One. 2026 Feb 18;21(2):e0342773. doi: 10.1371/journal.pone.0342773 (PMC12915946; doi:10.1371/journal.pone.0342773)
Supplement: Attachment — Submitted filename: Response_to_reviewers_auresp_3.docx [file pone.0342773.s005.docx]

Dear Editor,

Thank you for the opportunity to revise and resubmit our manuscript with the id PONE-D-25-25048R2 and altered title “Swedish private-sector employees’ experiences of promoting and hindering factors for working while having mental health problems: a qualitative study”. We would also like to thank you and the reviewers for your valuable comments. We believe these have contributed to improving our manuscript. Our point-by-point responses are given below. The changes described here are also highlighted in yellow in the manuscript.

On behalf of the authors,

Yours sincerely,

Anna Frantz

Thank you for your efforts in revising the manuscript in accordance with the reviewers’ comments. However, the Introduction and Methods sections still require further strengthening. In addition, the manuscript needs overall English proofreading.

Author response: Thank you for your encouraging words! In addition to revising the manuscript, we followed your advice on English proofreading. The current version has now undergone a thorough English language review by a professional language editor.

**Additional Editor Comments:**

The manuscript needs overall English proofreading. For example, the phrase “for working with mental health problems” may be better expressed as “when working while having mental health problems.”

Author response: Thank you for this suggestion. The manuscript has now undergone a thorough English language review by a professional language editor. In addition to revising the wording in accordance with your suggestion above, minor changes have been made throughout the manuscript to increase clarity.

Introduction
The Introduction and the study objectives remain insufficiently clear.
As the objectives are clearly articulated in the abstract, I recommend incorporating the same level of clarity into the Introduction.
Please further develop lines 81–86 on page 5.
The concept of presenteeism appears abruptly without any supporting rationale, which makes it difficult for readers to understand.

Author response: Thank you for these suggestions. We have revised the introduction to clarify the study rationale. We have also elaborated on the presenteeism concept. We agree that it appeared abruptly and we have moved the section on presenteeism to enhance the flow in the introduction.

“Working while having MHPs can be conceptualized through presenteeism – the behavior of going to work while experiencing ill-health [4]. Previous research has studied the potential negative impact of presenteeism on work productivity and employee health [4, 5]. However, presenteeism has also been suggested to include potential functional or therapeutic aspects, such as being an individual strategy to maintain or regain function [6]. In this study, in line with this conceptualization, working while having mental health problems (presenteeism) is therefore seen as a behavior that may have negative or positive implications [4].

Methods
The criteria for participant selection are unclear.
When recruiting workers experiencing MHPs, please specify which conditions are included under MHPs and clarify whether these were medically diagnosed or self‑reported.

Author response: We have clarified the criteria for selection in the first paragraph under the heading “Participants and procedures”.

“Employees at risk of sickness absence due to MHPs were identified through self-reports scoring with a cut-off of ≥3 points on the General Health Questionnaire 12-item (Swedish version) validated in the general population to detect depression [24], or answering ”yes, most likely” or ”yes, quite likely” to a question on self-rated risk of sickness absence due to MHPs in the year to come (i.e. “About your health – do you think you will receive sick leave benefits because of stress, anxiety or depression in the coming 12 months?”. The response options were “Yes, most likely”,” Yes, quite likely”, “I’m not sure”, “No, probably not”. Further inclusion criteria were 18–59 years of age, ability to understand written and spoken Swedish.”

You also mention presenteeism criteria, but additional details are needed, such as the name of the instrument used and whether criteria (e.g., at least 3 out of 12 items) were applied.

Author response: Thank you for this comment. Presenteeism was included as a theoretical construct to describe the behavior of working while ill, but we did not include any instrument as selection criteria for the sample. The inclusion criteria were based on risk for sickness absence due to mental health problems operationalized as stated above. We therefore consider the sample to be at work while also having mental health problems.

Furthermore, while the Methods section describes data collection and analysis procedures, it does not provide the main interview questions or follow‑up probes.
These should be presented for clarity.

Author response: We agree that presenting the questions and probes within the article could add clarity. Sample questions from the interview guide have been added to the manuscript in the first paragraph under the heading “Data collection” with referral to the full interview guide as supporting information (S1 Appendix. Interview guide).

“The interview started with a broad, open question: “Tell me about your work. What does a normal working day look like?” Examples of questions to follow were “If you think about the workplace, was there anything that made it easier to stay at work?” and “Did you use any specific strategies to be able to stay at work?”. Prompts were used to encourage elaboration, e.g., “Could you give an example of a situation?” or “Could you tell me more about that?”. The full interview guide is provided in S1 Appendix.”

Reviewers' comments:

Reviewer #1: Thank you for your careful revisions and for addressing the previous comments in depth. The manuscript has improved substantially in clarity, methodological transparency, and conceptual consistency. Your refinements—particularly regarding terminology, reflexive thematic analysis, and the elaboration of limitations—have strengthened the scientific rigor of the study. The manuscript is now very close to being ready for publication.
Below are a small number of minor suggestions that would further improve clarity and coherence.

Major (Minor) Points
1. Clarify the research gap more explicitly in the Introduction.
Although the Introduction has been improved, adding one or two sentences that clearly state why private-sector, low-skilled employees are underrepresented in prior qualitative research would strengthen the rationale for the study.

Author response: Thank you for this suggestion! We have revised the introduction to clarify the rationale for the study, with the beforementioned clarification of presenteeism, and a focus on the workplace as an arena for qualitative research on hindering and promoting factors for working while having mental health problems.

“In Sweden, employers have the responsibility to systematically work to reduce risks that can lead to work-related injury or ill-health, including MHPs [17]. Hence, the workplace provides an arena for preventing sickness absence due to MHPs [18] and understanding what promotes or hinders working while having MHPs could elucidate not only individual strategies, but also factors at a group, leader, or organizational level.”

2. Add a brief statement linking Table 2 to the Results section.
Table 2 is useful, but including a sentence early in the Results to highlight that the table summarizes promoting and hindering factors across the themes would help guide readers.

Author response: We have added a sentence to the first paragraph of the Results section referencing Table 2.

“An overview of the promoting and hindering factors is presented in Table 2.”

3. Ensure conceptual consistency in the use of “presenteeism.”
Since you have moved away from “stay at work,” a brief explanation in the Introduction about why “presenteeism” is used as a neutral behavioral concept would strengthen coherence across sections.

Author response: We have clarified the use of the presenteeism concept in the introduction, at the end of the first paragraph.

“Working while having MHPs can be conceptualized through presenteeism – the behavior of going to work while experiencing ill-health [4]. Previous research has studied the potential negative impact of presenteeism on work productivity and employee health [4, 5]. However, presenteeism has also been suggested to include potential functional or therapeutic aspects, such as being an individual strategy to maintain or regain function [6]. In this study, in line with this conceptualization, working while having mental health problems (presenteeism) is therefore seen as a behavior that may have negative or positive implications [4].

4. Reflexive thematic analysis: further clarification.
Add one sentence explaining how coding discussions enhanced reflexivity (e.g., surfacing assumptions and broadening interpretation).

Author response: We have reviewed the formulation around reflexivity in the coding process and added a sentence in the second paragraph of the Methods section on how discussions enhanced reflexivity.

“To enhance reflexivity, differences in coding and assumptions on the meaning of the data were discussed between AFr and EBB. These discussions helped bring assumptions to the surface and broaden the interpretation of the data.”

Consider adding a brief note on researcher positionality to increase transparency.

Author response: To increase transparency, ‘Notes on authors’ have been added at the end of the manuscript.

**Notes on authors**

Anna Frantz is a PhD student at Karolinska Institutet. She is also a licensed physiotherapist. Her PhD project focuses on sickness absence due to mental health problems with both a rehabilitative perspective in a primary healthcare setting, and a secondary preventive perspective among private-sector employees.

Iben Axén, PhD, licensed chiropractor, is an Associate Professor in Musculoskeletal Health with a specific interest in back pain and musculoskeletal disorders. She also has an interest in intervention development and conduct.

Gunnar Bergström, PhD, Professor in Occupational health conducts research in areas such as stress and mental ill health in working life, the impact of sick leave on health and work performance, being at work despite health problems, and the importance of the organizational and social work environment for health and illness.

Anna Finnes, PhD, licensed Psychologist. Anna’s research includes developing and evaluating interventions for mental health problems with a specific return to work-focus.

Elisabeth Björk Brämberg, PhD, Reg. Nurse, is Associate Professor in Occupational Medicine at Karolinska Institutet and senior lecturer in Insurance Medicine at the University of Gothenburg. Her expertise covers quantitative and qualitative methods. She has extensive experience of intervention development with prevention of sick-leave and return-to-work focus.

5. Clarify the classification of physical work environment factors.
A short explanatory sentence in the Discussion (e.g., that cold/noisy environments are organizational-level factors within the IGLOO framework but relate specifically to physical working conditions) would prevent confusion.

Author response: Thank you for this suggestion. We have added a sentence referencing the organizational level of the IGLOO framework in the fourth paragraph of the discussion section.

“The physical work environment related to the organizational level in the IGLOO framework, (e.g., noise and cold temperatures) was identified as a hindering factor for working while having MHPs”

Minor Points
・Check for minor inconsistencies in terminology (e.g., “first-line manager” vs. “first line manager”).
・Consider briefly clarifying that “life-stage” in Theme 1 includes both family responsibilities and career stage.
・Conduct a final check for consistent citation formatting.

Author response: We have conducted an English language review by a professional language editor, checking for inconsistencies throughout the manuscript. We have also clarified that theme 1 includes both private- and work-life domains (in the Results section and the Discussion). We have also checked the citation formatting for consistency.

Your manuscript is strong, relevant, and well developed. The remaining issues are minor and can be addressed with modest revisions. I look forward to seeing the revised version.

Reviewer #2: Thank you for giving the opportunity to review the revised manuscripts.
I have no additional comments, same as the first round.
